# The Effect of Scuba Diving on Microleakage of a Class II Composite Restoration: An In-Vitro Study

**DOI:** 10.3390/healthcare9060768

**Published:** 2021-06-19

**Authors:** Maryam Shahnavazi, Behzad Salari, Reza Fekrazad

**Affiliations:** 1 Department of Oral and Maxillofacial Radiology, School of Dentistry, Aja University of Medical Sciences, Tehran 3413941661, Iran; maryamshahnavazi@gmail.com; 2 Department of Orthodontics, School of Dentistry, Aja University of Medical Sciences, Tehran 3413941661, Iran; 3 Radiation Sciences Research Center, Laser Research Center in Medical Sciences, Aja University of Medical Sciences, Tehran 3413941661, Iran; rezafekrazad@gmail.com

**Keywords:** dental barotruama, air pressure, diving, dental leakage, composite restoration

## Abstract

Persistent pressure change is a common phenomenon within scuba diving with various medical and dental sign side effects. This study evaluates the effect of simulated pressure change due to scuba diving on the microleakage of class II composite restoration. In our methodology, a total number of 150 intact bicuspids are divided into two main groups (A and B), and prepared for a class II composite restoration. The samples of each main group are divided into five subgroups to be prepared with different liners. Then samples are restored with the same resin composite material. The teeth in group A are thermocycled under the normal atmospheric pressure, while group B are thermocycled under simulated scuba diving conditions. The gingival microleakage is assessed based on dye penetration. The group B teeth show a significantly higher microleakage score than their equivalents in group A (*p* < 0.05). The subgroups without a liner have a higher microleakage score than the other subgroups (*p* < 0.05). The flowable composite shows the leased leakage scores followed by Nano ionomer, Resin Modified Glass Iononomer, GIOMER, and linerless groups (*p* < 0.05). Scuba diving could increase the risk of microleakage development beneath class II, a composite restoration.

## 1. Introduction

Scuba diving is a kind of diving in which scuba is used to make a diver independent from surface oxygen to breathe [1]. The total pressure a diver experiences at a certain depth is the sum of water and air pressure. Therefore, the more depth a diver descends, the more pressure he or she suffers. Dental barotrauma is a term referred to the side effects of pressure changes on teeth, including failure of dental restorations, tooth fracture (barodontocrexis), or tooth pain (barodontalgia) [2,3]. It was reported that the dental side effects of scuba diving occur at diving depths of 10–25 m, a common depth for scuba diving [4]. Goethe et al. [5], in a 10-year prospective study on the military divers who tolerated constant barometric changes, reported a 4-fold increase in the amount of extracted teeth and a ten-fold increase of teeth that need a prosthetic crown. Calder and Ramsey [6] attributed leakage as the main cause of restoration failure in the rapid decompression condition. Furthermore, Shafigh et al. [7] revealed a non-significant difference between microleakage of composite and amalgam restorations in the simulated diving condition.

Polymerization shrinkage is a physical phenomenon in which a microgap is formed between the tooth structure and restorative material. This gap is a potential place for developing secondary caries, hypersensitivity, and color change which could clinically decrease the durability of composite restorations. Due to higher occlusal forces and harder accessibility, polymerization shrinkage has been an important consideration for restorations of posterior teeth, especially at the gingival margin of class II cavities [8]. Changing resin-base formulation, manipulating different insertion techniques, and reducing restorative bulk using different liners below main restorative material has been proposed to decrease polymerization shrinkage [9]. Glass ionomer luting has a similar coefficient of thermal expansion to dentine. Therefore, applying this material as a liner could decrease gap formation caused by the difference between this coefficient in two surfaces, due to persistent thermal expansion and constriction of teeth in the oral cavity [10]. Flowable composites have a lower modulus of elasticity than bulk composites; therefore, applying this material as a liner could compensate for the polymerization stresses and decrease gap formation below class II composite restorations [11].

As described before, it was suggested that rapid pressure change within scuba diving could create internal tensions within structural cracks of teeth or the gaps between a tooth structure and a restoration. This phenomenon is attributed to the inability of the environment to adjust the internal and external pressure, due to rapid change. We assumed that the persistent repetition of this action in a long time might enlarge the micro-gaps created beneath a class II composite restoration. We found few well-designed studies which evaluate the microleakage of direct restorations in different ambient pressures. In this study, we aimed to evaluate the effect of persistent pressure change and different lining materials on the microleakage of a common class II composite restoration.

## 2. Material and Methods

### 2.1. Tooth Selection and Preparation

**Figure 1:**
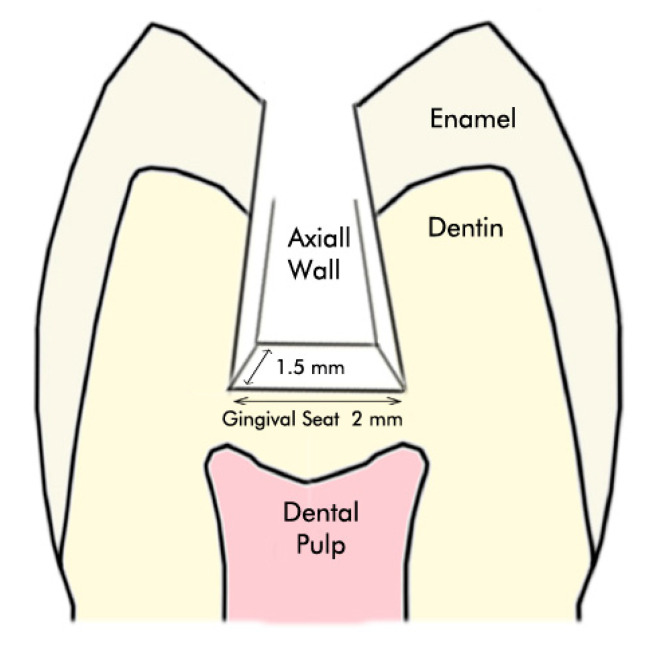
The prepared proximal box from the mesial view.

The present study was approved by the Ethics Committee of AJA University of Medical Sciences (code no. 423721) and designed based on the previous investigations [12,13]. The sample size calculation was done considering the use of two-tailed statistics with 80 percent power and an alpha level of 5 percent. One hundred and fifty extracted maxillary bicuspids were obtained. None of them had caries, cavities, or dental restorations. All the samples were extracted for orthodontic purposes. Their surface debris was cleaned with an ultrasonic device and stored in normal saline. For disinfection purposes, two weeks before preparation, all of them were immersed in a 0.5% chloramine T trihydrate solution. After that, class II cavities were prepared on the mesial surface of bicuspids using straight and pear-shaped diamond burs (Diatech Dental AG, Heerbrugg, Switzerland) and a high-speed handpiece with water-cooling. The prepared cavity had 2 mm width buccolingually and 1.5 mm depth pulpally (Figure 1). The gingival seat of the proximal box was placed in the enamel and 1 mm above the cementoenamel junction (CEJ). A digital caliper (Mitutoyo 500, Mitutoyo Corporation, Kanagawa, Japan) was used to ensure uniformity among preparations. All the preparations were done by the same operator (an expert restorative dentist) to eliminate bias and maintenance of blindness. The diamond burs were renewed after every five preparations, to eliminate thermal side effects on the tooth structure.

### 2.2. Restorative Procedure

After cavity preparation, teeth were randomly divided into two main groups of A and B by another operator (for the blindness purpose). After that, the prepared teeth in\group A were randomly divided into five subgroups (A1 to A5), each contained 15 specimens and treated as follow:

Subgroup A1: The specimens were etched with 35% phosphoric acid gel for 15 s, rinsed, and dried with absorbent paper. Single Bond II (3M ESPE, St. Paul, MN, USA) was applied in two layers with disposable applicators, each one was dried for five seconds for solvent evaporation, and light cured with an LED source according to the manufacturer’s instructions. Light curing procedure was done by the Soft Start mode, which began with the intensity of 200 mW/cm^2^ up to 800 mW/cm^2^ (Demi LED Light Curing System, Kerr Corp, Orange, CA, USA). We did not add any material as a liner on the prepared boxes.

Subgroup A2: The specimens were etched with 35% phosphoric acid gel for 15 s, rinsed, and dried with absorbent paper. The bonding agent was applied the same as the specimens of Subgroup A2 and light cured with an LED source according to the manufacturer’s instructions. A thin layer of GIOMER (Beautifil Flow Plus F03, Shofu, Kyoto, Japan) was used as a liner in the axiogingival line angle between axial and gingival floors. The closed sandwich technique was used for liner placement. The thickness of the liner was within 0.5 to 0.7 mm. GIOMER placed 0.8 mm away from the cavosurface margins, and then be light cured as previously discussed.

Subgroup A3: In these specimens, Nano-Ionomer was used. The gingival surface of prepared boxes was pretreated with Ketac N100 Nano Ionmer Primer (3M ESPE, St. Paul, MN USA). Then Ketac N100 (3M ESPE, St. Paul, MN, USA) was hand mixed and prepared according to their manufacturer’s recommendations. It is applied as a liner and light cured with an LED source for 20 s. The lining steps were taken in the same way as described in subgroup A2.

Subgroup A4: The specimens were etched with 35% phosphoric acid gel for 15 s, rinsed, and dried. The Single Bond (3M ESPE, St Paul, MN, USA) was applied and light cured using an LED source according to the manufacturer’s instructions. A flowable composite (Filtek Flow, 3M ESPE, St.Paul, MN, USA) was used as a liner in the prepared teeth of this subgroup. The steps of the liner application were similar to the specimens of subgroup A2.

Subgroup A5: A resin modified glass ionomer (RMGI) cement (Fuji II LC, GC Corp, Tokyo, Japan) was prepared according to the manufacturer’s recommendations and used as a liner in these specimens. The 10% Polyacrylic Acid was applied for 20 s as a dentin conditioner (GC Corp., Tokyo, Japan), then rinsed and air-dried. The lining steps were performed in the same way as mentioned for subgroup A2.

Following the above mentioned steps, the restorative process was done for all teeth. A metal band and Tofflemire matrix holder were placed, and constant finger pressure against the gingival margin was applied to prevent overfilling in the gingival margin. The first 1-mm layer of restorative composite (Filtek P60, 3M ESPE, St. Paul, MN, USA) was placed horizontally and adapted to the proximal box; then it was light cured for 40 s. The remaining three layers were inserted obliquely and light cured separately for 40 s. Each oblique layer had 2 mm thicknesses. The band was then removed, restoration was light cured for 20 s, and then a finishing procedure was done using a series of discs (Sof-Lex, 3M ESPE). No finishing procedure was done on the gingival margin of the final restoration.

In the second step, the other 75 prepared specimens (main group B) were randomly divided into five subgroups (B1 to B5); each contained 15 prepared teeth. The same allocation and nomination process, as well as restoring and finishing techniques, were done for these specimens. Following the abovementioned steps, the specimens of both groups were stored in the deionized water in a sealed container at 37 degrees centigrade for 24 h.

### 2.3. Thermo Cycling and Scoring Process

The stored teeth in group A, were thermocycled at normal atmospheric pressure at sea level (1 bar) and 5–55 degrees centigrade for 1000 cycles with a dwell time of half a minute in each bath and 1/3 min interval between baths. The specimens of group B were thermocycled under a simulated diving condition. The simulator was a customized pressure chamber that was programmed to change internal pressure between 1 to 4 bar. A thermocycling machine (SD Mechatronik, Feldkirchen-Westerham, Germany) was placed within the pressure chamber to simulate the oral envoiroment. While the specimens were thermocycling (between 5–55 degrees centigrade for 1000 cycles with a dwell time of half a minute in each bath and 1/3 min interval between baths), the pressure in the simulator pot was changing. The simulator was programmed to incrementally increase the ambient pressure from 1 to 4 bar, and then is decreased from 4 to 1 bar with a dwell time of 20 s for each pressure. The stimulation procedure was finished after the termination of thermocycling cycles. This procedure was designed to create a simulated condition that a professional scuba diver might experience [14]. Following thermocycling, the specimens of both groups were coated with two layers of fingernail varnish, extending 1 mm beyond the margins of restorations. The apexes of specimens were sealed by special wax. After the sealing procedure, all teeth were immersed in 2% methylene blue for 24 h. Then, they were completely rinsed and cleaned with deionized water. Each tooth was sectioned mesiodistally across the center of the restorations using a diamond disk (IsoMet, Buehler Ltd., Lake Bluff, IL, USA). The sections were evaluated under a stereomicroscope at 40× magnifications (Olympus SZ 61, Olympus Corporation, Shinjuku-ku, Japan) to score the dye penetration by another examiner (an expert pathologist). The scoring procedure was blindly repeated one day later by the same examiner. The final score was gathered using the worst score collected for each specimen.

Score 0: No die penetration

Score 1: Penetration to half of the gingival wall

Score 2: Penetration to more than 1/2 the gingival seat

Score 3: Penetration to the full extent of the gingival wall, excluding the axial wall

Score 4: Penetration to the full extent of the gingival wall, including the axial wall

### 2.4. Statistical Analysis

The microleakage scores were statistically analyzed using a non-parametric one-way analysis of variance (Kruskal-Wallis) test and Mann–Whitney U-test. Statistical significance was set in advance at the 0.05 confidence level. Kruskal-Wallis test was applied to compare all the subgroups in each environment, and Mann–Whitney U-test was applied to compare each subgroup with its counterpart in the other environment (A1–B1, A2–B2, …).

## 3. Results

**Figure 2:**
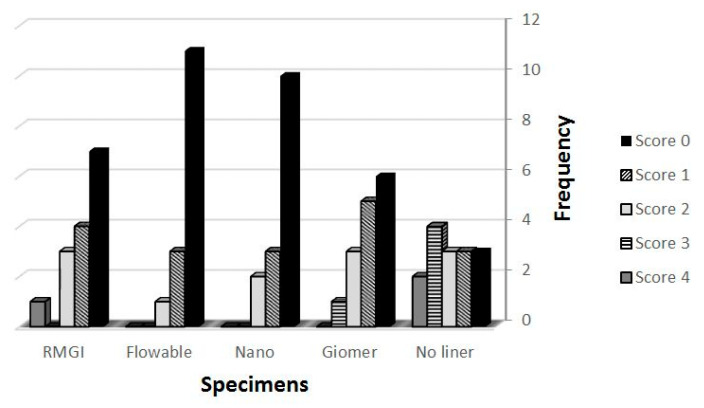
The frequency of microleakage scores in the specimens of group A.

**Figure 3:**
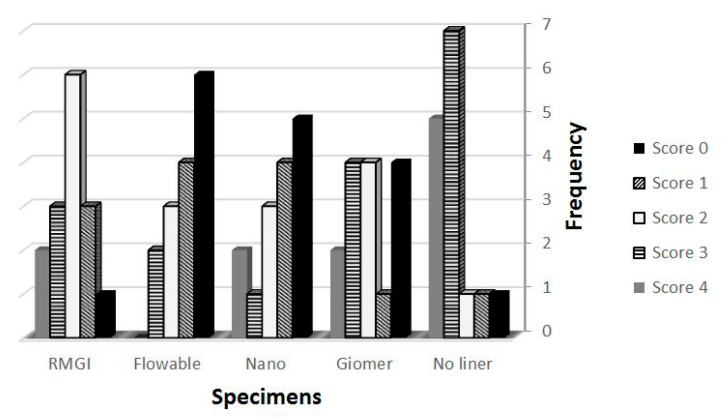
The frequency of microleakage scores in the specimens of group B.

The specimens which were thermocycled under persistent pressure change (group B) showed significantly more leakage score than their counterparts in group A which were thermocycled under normal atmospheric pressure (Table 1). There was a statistically significant difference between group A (no liner) and its subgroups (*p* < 0.05) and group B (no liner) and its subgroups (*p* < 0.05). According to the mean microleakage score of the specimens, the flowable composite in both main groups showed the leased leakage scores followed by Nano ionomer, Resin Modified Glass Iononomer, GIOMER (Figure 2 and Figure 3). The specimens without liner in both groups (A1, B1) showed the most leakage scores (*p* < 0.05).

## 4. Discussion

Due to the increased tendency to scuba diving among the young population who are seeking adventure, oral health keepers will increasingly encounter signs and symptoms of barodontalgia. The law of Boyle–Mariotte, could explain some of these side effects, it reveals “at a constant temperature the volume and pressure of an ideal gas are inversely proportional”. This phenomenon could result in pain and fracture of displacement of dental restorations [2]. This study focused on another aspect and evaluated the effect of pressure change on the gingival microleakage of a class II composite restoration.

The results showed that applying any liner with a closed sandwich technique decreased the microleakage either in the normal ambient pressure or simulated diving condition. The specimens restored without liner had more microleakage compared with other groups. Similar to this finding, Darsan et al. [15] showed closed sandwich technique significantly reduces microleakage. They also reported that the specimens restored without liner had the highest microleakage. Von Fraunhofer et al. [16] reported higher microleakage scores for the specimens restored with liner and/or base. This difference could be because of the different tooth preparation methods in which class V cavities were made. Moreover, the electrochemical-induced leaking process in this study might affect the results. In both environments, the flowable composite was more effective than other liners. Some authors proposed that the elasticity of flowable composite helps it act as a stress-resorptive intermediate layer beneath the main restoration [17]. They reported that the presence and thickness of flowable composite could influence the magnitude and the direction of the shrinkage. This intermediate layer mainly influences the magnitude, and to a certain extent, the direction of the shrinkage vectors. Stefanski et al. [18] reported the lower modulus of elasticity helps flowable composite to be more effective than the other liners. However, some authors declined the ability of this material to decrease the shrinkage stresses that cause cuspal deflection [19]. It was proposed that flowable composite provide a fine gingival adaptation and decrease dye penetration as a liner even in the deep class II preparations [20]. In contrast, Braga et al. [21] reported that applying a flowable composite as an intermediate layer did not decrease the gingival microleakage. This controversy may result from the different methods in which the flexural strength and contraction stress were evaluated based on the computerized model, not in-vitro assessment.

Majety et al. [22] reported that applying flowable composite as a liner could improve the sealing ability of composite restorations. Moreover, the RMGI showed a lower sealing ability than flowable composite in different thicknesses. The authors attributed this difference to the structural micro-gaps that originated from the particle size and viscosity of RMGI. However, some authors reported controversial results that RMGI is more effective than flowable composite [23]. The different preparation techniques in which the gingival seat of proximal boxes was located 1 mm below CEJ in dentin may cause this difference. In the current study, the gingival margin was located above CEJ in enamel.

RMGI has a compatible coefficient of thermal expansion with tooth structure, and a slow and continuous setting reaction that helps absorb local stresses [24,25]. The chemical bond between RMGI and tooth structures is more resistant against water penetration than that established between a resin-based dental material, such as GIOMER. This might explain the lower leakage score. In the current study, this score was lower for flowable composite than RMGI, which could be attributed to the higher bond strength between flowable composite and enamel. Most of the previous studies evaluated the microleakage of nano-ionomer in the class V cavities. El-Ashiry et al. [26] reported that the sandwich technique with nano-ionomer is a proper method to decrease microleakage in class V cavities of primary molars. This effectiveness is attributed to the improved sealing ability and decreased contraction stresses, due to the high filler content and low coefficient of thermal expansion.

GIOMER showed the highest microleakage scores among subgroups that restored with a liner. Similar to this finding, Ab Malik et al. [27] demonstrated that flowable composite had a lower microleakage than GIOMER. But some authors showed that GIOMER had a higher microleakage than an RMGI. The class V cavity and different box preparation may affect this difference [28]. The increased microleakage of this material is attributed to the hygroscopic expansion phenomenon in this material [29]. Marinova-Takorova et al. [30] reported that GIOMER had higher microleakage than control groups. But they applied GIOMER as a restorative material, not an intermediate layer beneath the main restoration.

The normal air pressure at sea level is equal to 1 bar. For every 10 m of diving, the pressure increases 1 bar. Therefore, the body of scuba divers is exposed to 2 bars at a depth of 10 m, 3 bars at a depth of 20 m, and 4 bars at a depth of 30 m. We noticed that the specimens of all subgroups showed significantly more microleakage and die penetration score under persistent pressure change compared with the specimens which were evaluated under the normal atmospheric pressure. According to Boyle’s Law, “the volume of gas at constant temperature varies inversely with the surrounding pressure”; therefore, the persistent pressure during professional scuba diving could affect the volume of gas trapped in the cavities or small voids under composite restorations.

Some authors reported that pressure change cycles increased microleakage in the class II composite restorations [30]; although the differences were not statistically significant. In this study, the sandwich technique was not applied, and the pressure change cycles were set according to the simulation of the flight and scuba diving. Safaie et al. [31] reported that increasing the atmospheric pressure could slightly increase dye penetration through the root canal system in different obturation methods. They proposed that according to Boyle’s law, we could minimize the adverse effect of pressure changes by decreasing void formation within root canal fillings. Goethe et al. [5] performed a prospective study on the expert navy divers. They indicated a ten-fold increase in the crown removal of divers. The authors attributed the results to the barometric changes that those divers experienced. The increased microleakage in the group B teeth may originate from the pressure-related tensions. Those created due to volumetric changes in gases trapped under the restorations. These continuous tensions could weaken marginal integrity or deepen the micro-gap (increase the microleakage score). In contrast to the current results, Shafigh et al. [7] demonstrated no significant difference between microleakage of composite and amalgam restorations in different pressure. They evaluated the microleakage score in two different pressures (0.5 and 2 bar), whereas we assessed it under a simulated diving condition (persistent pressure change between 1 to 4 bar).

Although the experimental conditions of this study did not fully simulate the oral environment of a scuba diver and factors, such as oral hygiene, diet, and tooth mineralization, could affect the durability of composite restorations [32], the current findings had important clinical outcomes. The restored teeth in the simulated scuba diving condition showed significantly higher gingival microleakage. Moreover, applying the liner according to the closed sandwich technique decreased dye penetration in both environments. Further well-designated studies with a larger population, more accurate simulations, and application of recent technologies in digital dentistry [33] could decrease the errors and reveal more accurate information to clarify this topic.

## 5. Conclusions

Within the limitations of this study, we have concluded that pressure change cycles within scuba diving (1 to 4 bar) increased gingival microleakage of a class II composite restoration. Application of a liner (flowable composite, RMGI, Nano-ionomer, and GIOMER) according to the closed sandwich technique, significantly decreased microleakage in both environments (simulated scuba diving condition and ambient pressure). The flowable composite showed the lowest microleakage, followed by Nano ionomer, Resin Modified Glass Iononomer, GIOMER. The teeth restored without liner had highest microleakage in both environments.

## Figures and Tables

**Table 1:** Comparison of microleakage score between counterpart subgroups using Mann–Whitney test.

Liner	Environment (Subgroup)	Score (Mean)	*p*-Value
No Liner	Scuba Diving (A1)	12.30	0.039
	Ambient Pressure (B1)	18.70	
GIOMER	Scuba Diving (A2)	12.43	0.049
	Ambient Pressure (B2)	18.57	
Nano-Ionomer	Scuba Diving (A3)	12.47	0.043
	Ambient Pressure (B3)	18.53	
Flowable Composite	Scuba Diving (A4)	12.57	0.042
	Ambient Pressure (B4)	18.43	
Resin Modified- Glass Ionomer	Scuba Diving (A5)	11.23	0.006
	Ambient Pressure (B5)	19.77	

## Data Availability

Data supporting reported results will be available by contacting the corresponding author. B.salarii@gmail.com.
